# Supplementary material for: Splice-Junction-Based Mapping of Alternative Isoforms in the Human Proteome
Source: Cell Rep. Author manuscript; Available in PMC 2020 Jan 15. (PMC6961840; doi:10.1016/j.celrep.2019.11.026)
Supplement: 2 [file NIHMS1546469-supplement-2.zip › DF1/PXD010154/Thyroid/Thyroid_5_TG_NSDMGSRPESMGCR.pdf]

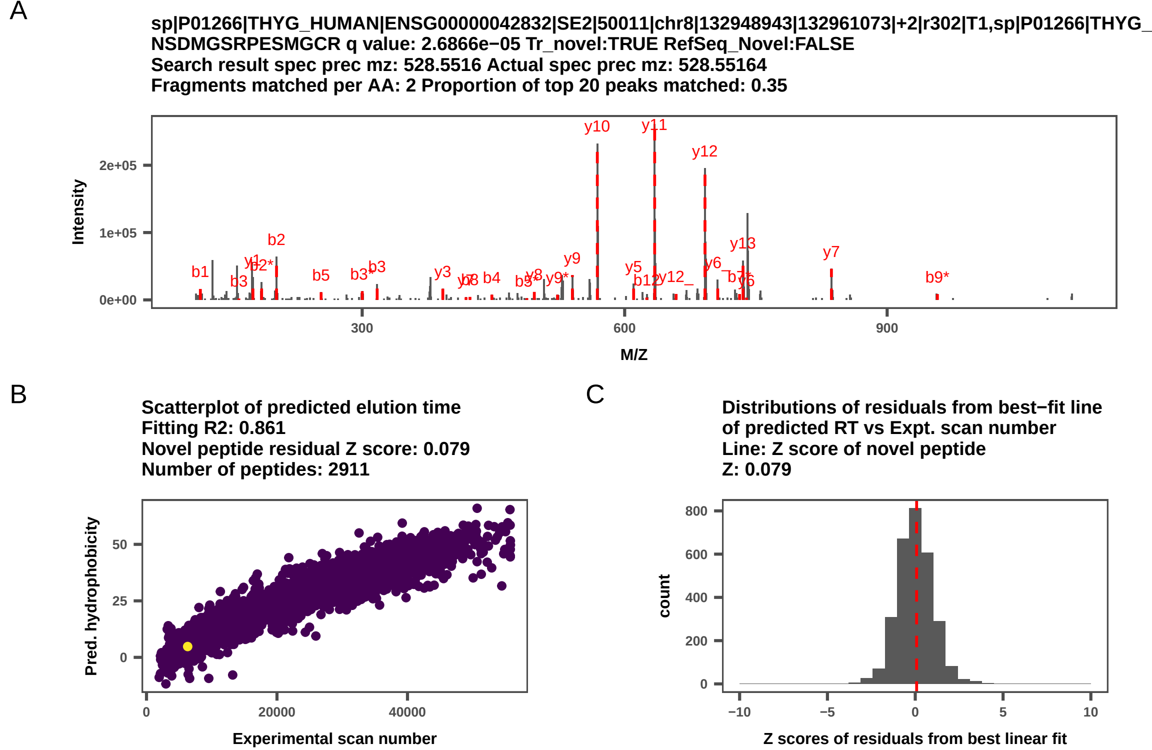
A
sp|P01266|THYG_HUMAN|ENSG00000042832|SE2|50011|chr8|132948943|132961073|+2|r302|T1,sp|P01266|THYG_
NSDMGSRPESMGCR q value: 2.6866e−05 Tr_novel:TRUE RefSeq_Novel:FALSE
Search result spec prec mz: 528.5516 Actual spec prec mz: 528.55164
Fragments matched per AA: 2 Proportion of top 20 peaks matched: 0.35
Intensity
2e+05
1e+05
0e+00
y10
y11
y12
y13
y7
b9*
b2
b1
b3
b5
b3*
y3
b4
y9
y5
y6
300
600
900
M/Z
B
Scatterplot of predicted elution time
Fitting R2: 0.861
Novel peptide residual Z score: 0.079
Number of peptides: 2911
Pred. hydrophobicity
50
25
0
0
20000
40000
Experimental scan number
C
Distributions of residuals from best−fit line
of predicted RT vs Expt. scan number
Line: Z score of novel peptide
Z: 0.079
count
800
600
400
200
0
−10
−5
0
5
10
Z scores of residuals from best linear fit
